# Engineering microbial consortia by division of labor

**DOI:** 10.1186/s12934-019-1083-3

**Published:** 2019-02-08

**Authors:** Garrett W. Roell, Jian Zha, Rhiannon R. Carr, Mattheos A. Koffas, Stephen S. Fong, Yinjie J. Tang

**Affiliations:** 1 Department of Energy, Environmental and Chemical Engineering, Washington University, Saint Louis, MO 63130 USA; 2 Department of Chemical and Biological Engineering, Rensselaer Polytechnic Institute, 110 Eighth Street, Troy, NY 12180 USA; 3 Department of Chemical and Life Science Engineering, Virginia Commonwealth University, Richmond, VA 23284 USA

**Keywords:** ^13^C-metabolic flux analysis, Cross-feeding, Metabolite channeling, Reporter protein, Subpopulations

## Abstract

During microbial applications, metabolic burdens can lead to a significant drop in cell performance. Novel synthetic biology tools or multi-step bioprocessing (e.g., fermentation followed by chemical conversions) are therefore needed to avoid compromised biochemical productivity from over-burdened cells. A possible solution to address metabolic burden is Division of Labor (DoL) via natural and synthetic microbial consortia. In particular, consolidated bioprocesses and metabolic cooperation for detoxification or cross feeding (e.g., vitamin C fermentation) have shown numerous successes in industrial level applications. However, distributing a metabolic pathway among proper hosts remains an engineering conundrum due to several challenges: complex subpopulation dynamics/interactions with a short time-window for stable production, suboptimal cultivation of microbial communities, proliferation of cheaters or low-producers, intermediate metabolite dilution, transport barriers between species, and breaks in metabolite channeling through biosynthesis pathways. To develop stable consortia, optimization of strain inoculations, nutritional divergence and crossing feeding, evolution of mutualistic growth, cell immobilization, and biosensors may potentially be used to control cell populations. Another opportunity is direct integration of non-bioprocesses (e.g., microbial electrosynthesis) to power cell metabolism and improve carbon efficiency. Additionally, metabolic modeling and ^13^C-metabolic flux analysis of mixed culture metabolism and cross-feeding offers a computational approach to complement experimental research for improved consortia performance.

## Background

**Fig. 1:**
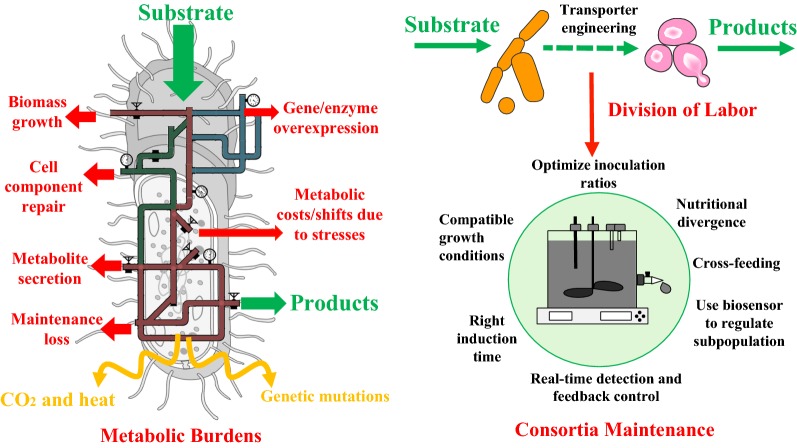
Overview of cellular processes, metabolic burdens, and resource allocations in engineered microbial hosts (left) and the consortia maintenance (right)

As the fields of synthetic biology have advanced, biological production has become a promising option for manufacturing various chemicals and pharmaceuticals. In principle, any compound with a defined synthesis pathway could be produced from cheap feedstock by an engineered microbial culture. Unfortunately, while recombinant microorganisms have been generated to produce some molecules, their productivity rarely reaches sufficient levels for commercialization [1]. One key barrier is metabolic burden (Fig. 1). Microbial hosts have to allocate limited resources amongst different tasks; this essential balancing act describes the key counterforce against any engineered pathway [2–4]. Additionally, chemical production and intermediate accumulation will often cause cell stress due to chemical toxicity [5], and the host may consume additional ATP/NADH (e.g., use of efflux pump) [6], leading to further resource shortages [7]. The synergistic combination of metabolic burden and cell stress leads to a deep drop in microbial biosynthetic performance, termed the “metabolic cliff” [4]. When the host hovers at the edge of this cliff, even small growth/stress perturbations can cause undesired metabolic responses and loss of production yields. To minimize such problems, Division of Labor (DoL) using microbial consortia becomes an alternative strategy [8]. Drawing inspiration from natural systems may provide one or more routes to circumvent this metabolic cliff. In higher organisms, specific metabolic tasks are partitioned off into membrane-bound organelles. Compartmentalizing different portions of a synthesis pathway can also serve to spatially separate mutually incompatible steps. From an ecological perspective, different species in microbial consortia cross-feed nutrients, allowing the system to achieve diverse functions from bioremediation to biochemical production [9, 10]. Even for genetically identical cells, multiple subpopulations can divide the biosynthesis labor or sequester toxic intermediates (as in heterocystous cyanobacteria). Extending these concepts to bioprocesses presents new opportunities for engineering pathways and controlling cell populations to maintain homeostasis (Fig. 1).

Microbial consortia can break up metabolic load among partners [11–13]. In the past, microbial consortia have been used for waste treatment, anaerobic digestion, the food/nutrient industry (e.g., dairy products, soy sauce, wines, and vitamins), as well as medical applications (e.g., gut microbiome). Recently, synthetic biology has successfully engineered co-cultures for the production of commodity chemicals (*cis*,*cis*-muconic acid) [14] as well as drugs (oxygenated taxanes) [15]. Synthetic consortia can be formed from several strains of the same species, such as engineered *E. coli* strains which together produce high-value chemicals such as flavan-3-ols [16], curcuminoids [17], and anthocyanins [18]. Current industrial applications of DoL using microbial consortia still face challenges in controlling population dynamics and optimizing productions. This review presents an overview of microbial consortia applications, limitations, and opportunities.

## Microbial consortia interactions, maintenance and stability

**Fig. 2:**
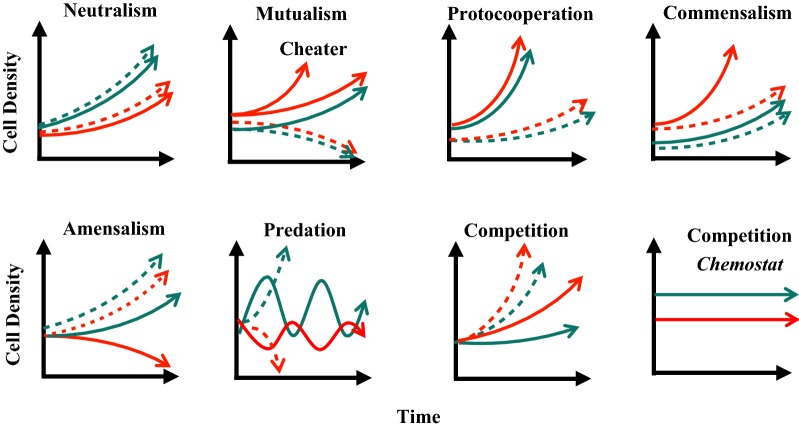
Interactions between two species in co-culture can have many different characters (red = species 1, green = species 2, solid = co-culture, dashed = pure culture)

Microbial communities are ubiquitous in natural environments and are key players in global carbon and nutrient cycles [19]. Several types of co-culture relationships are possible between two microbial species (Fig. 2) [20]. First, if two species consume different substances (i.e., nutritional divergence) and neither produces inhibitory compounds, the presence of each will not affect the other’s growth; this situation is described as ‘neutralism’. Second, if both species need the other to survive, which can happen when species mutually exchange required substances, or mutually remove toxins, the relationship is termed ‘mutualism’. As an example, a co-culture of *Desulfovibrio vulgaris* and *Methanococcus maripaludis* was developed for methane production from organic acids [19]. The paradigm for the syntrophic association is that methanogens create favorable thermodynamic conditions by scavenging hydrogen and keeping its partial pressure low, allowing the sulfate reducer to ferment carbon sources. Further, protocooperation is similar to mutualism, except that the interaction between species is beneficial to the growth rate of both populations but not required for either to live. Third, commensalism and amensalism both describe one-way interactions, where one species affects the well-being of another while remaining unaffected by its partner. In commensalism, the effect is beneficial, whereas in amensalism, growth of the affected species can be hindered due to the production of toxic compounds from its partner. Fourth, predation (or parasitism, less observed in microbial consortia) describe scenarios where one species’ growth depends on consuming the other, in which the population dynamics often show continuous oscillations. Finally, if different species compete for the same limited substrate, the faster-growing species will dominate over time. However, different species may still coexist at a stable population ratio in a chemostat culture when dilution rate and substrate concentrations are maintained at the crossover point where both species have the same growth rate [20].

Due to the complex interactions and dynamics of species within a community, consortia maintenance and stability is crucial for any successful applications (Fig. 1). Different microbes in consortia must grow in the same environment (temperature, media, pH, and oxygen) and the growth of one species must not destroy the other members in a short period. Since the growth rates of different species or different partner strains from the same species will not be identical, one species can eventually take over the culture. To balance the subpopulations, several approaches may be employed. First, the inoculation ratios for different partners must be optimized. Second, intermittent supplementation of underdog subpopulations may elongate the period of co-cultivation. With the aid of real-time detection systems [21], optimized population composition and bioprocess parameters can be closely monitored and maintained across the fermentation by feeding the desired cultures. Third, biosensors (e.g., quorum sensing through cell-to-cell communication) may potentially be used to control cell sub-population [22]. Fourth, cell immobilization can be attempted (e.g., growing free cells of *Pichia stipites* and immobilized *Zymomonas mobilis* together for ethanol production) [23]. Fifth, coexistence partners often compete for substrates, but nutritional divergence or syntrophy (one species lives off the products of another species) can be employed to avoid substrate competition. Such concepts have been widely applied for utilization of mixed substrates or cascade biodegradation of recalcitrant feedstock. Importantly, mutualistic growth is desirable for stable consortia applications. During this cooperation, a species benefits from the waste of another, while the waste producer might also receive costly resources in return. Such mutualistic consortia can show stable performance over prolonged cultivations [19]. Moreover, in situ evolution can improve the robustness of mutualistic systems. For example, in a stable co-culture, *Salmonella enterica* evolved to secrete methionine, a costly amino acid, for an *E*. coli strain, while the *E. coli* evolved novel secretion of sugar to feed *S. enterica* [24, 25].

## Applications of DoL for substrate utilizations

Microbial communities have been extensively studied for microbial ecology and waste treatment. Industrial biotechnology has applied the same principles to increase bioproduction from cheap feedstocks such as CO_2_ and cellulosic biomass. Synthetic consortia composed of bacteria and fungi are a particularly promising method for utilizing agricultural wastes to achieve waste-to-fuel/waste-to-food processes [26].

### Consolidated bioprocesses

**Fig. 3:**
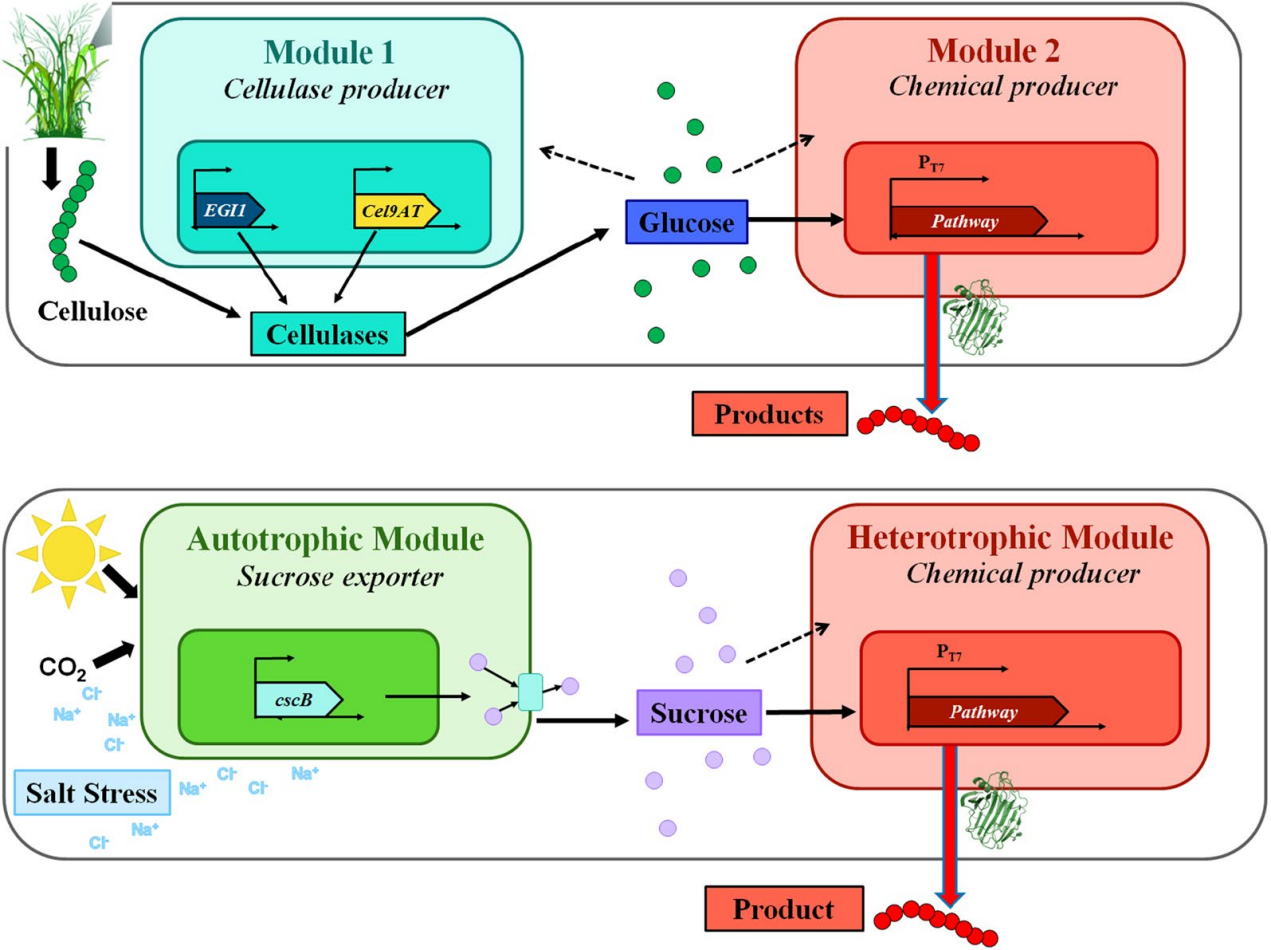
Co-culture for CBP. Above: using a cellulosic feedstock; bottom: using CO_2_

Consolidated bioprocesses (CBP) aim to directly convert lignocellulosic biomass to biofuel. Attempts have been made to engineer a super bug that can convert cellulose to ethanol or other fuels. In one attempt, *Saccharomyces cerevisiae* was engineered to carry mini-cellulosomes to give cells the ability to simultaneously break down and ferment cellulose to ethanol. However, this engineered strain only achieved a low titer of ethanol (~ 1.8 g/L) [27]. In contrast, cellulolytic thermophiles, such as *Clostridium thermocellum*, can be a CBP platform for ethanol production, but they have low ethanol yields and resistance to genetic modifications. To overcome drawbacks associated with single-organism CBP, synthetic co-cultures are developed, where cellulose is fed into the system and the product of interest is synthesized using two different organisms: a cellulose degradation module that secretes cellulases and a product synthesis module that consumes the resulting glucose to make the product (Fig. 3). For example, co-cultures of *Clostridium thermocellum* with bio-producing bacteria could be a promising approach. Specifically, the fermentation of cellulose and cellobiose by *Clostridium thermocellum* and *Methanobacterium thermoautotrophicum* co-culture was built to produce hydrogen gas, methane, acetic acid, and ethanol [28]. More recently, co-cultures of *Clostridium thermocellum* and non-cellulolytic *Thermoanaerobacter* strains significantly improved ethanol production by 4.4-fold compared to the monoculture [29]. CBP for butanol production from cellulosic biomass have been developed by taking advantage of the specific metabolic capacities of cellulosic *Clostridium celevecrescens* and butanol producer *Clostridium acetobutylicum*. They have achieved butanol concentration of 3.73 g/L [30]. Another avenue is to employ fungal strains paired with genetically-engineered *E. coli* for isobutanol production [31]. The fungus *Trichoderma reesei* secretes cellulases to hydrolyze lignocellulosic biomass and *E. coli* metabolizes soluble saccharides into isobutanol (achieving titers up to 1.9 g/L and yields up to 62% of theoretical maximum). *Trichoderma reesei* and *Rhizopus delemar* can also be coupled to convert cellulose to fumaric acid (6.87 g/L and 0.17 w/w yield) without supplemental cellulase enzymes [32]. In another example, a strategy was developed by constructing a cell-surface displayed consortium using two engineered yeast that heterologously expressed functional lignocellulolytic enzymes to convert pretreated corn stover to ethanol [33].

### Mixed sugar fermentations

For cellulosic feedstock, xylose derived from the hemi-cellulose component cannot be used by model yeast platforms. To address this, a xylose-fed co-culture of *E. coli* and *Saccharomyces cerevisiae* has been developed, wherein *E. coli* (which can naturally use xylose) generate acetate as waste product; *S. cerevisiae* consumes the acetate and reduces its inhibition of *E. coli* growth [15]. Similarly, consortia with different engineered *S. cerevisiae* strains can ferment glucose–xylose–arabinose mixtures [34].

### C1 carbon utilizations

Consortia can serve as platforms for utilization of C1 substrates (such as methane, CO_2_, and CO). For example, *Citrobacter amalonaticus* can assist *Sporomusa ovata* to produce acetic acid from carbon monoxide [35]. Moreover, methane is an abundant feedstock from anaerobic digestion or oil reservoirs. Archaeal–bacterial symbiosis could couple methane oxidation with sulphate reduction in gas-hydrate-rich sediments [36], which makes the anaerobic conversion of methane into bio-products possible. Among C1 substrates, biosequestration of CO_2_ has great potential. Algae, including both prokaryotic cyanobacteria and eukaryotic microphytes, can fix inorganic carbon for bio-production. However, algal growth and genetic tool development have lagged far behind that of classic heterotrophic hosts like *E. coli* and *S. cerevisiae* [37]. It is possible, however, for the autotrophy of microalgae to be exploited more indirectly, in a modular system that supplies organic carbon for a chemical-producing heterotrophic host (Fig. 3). Such modularity (algae-bacteria system) has been laid in the development of a sucrose-exporting strain of the cyanobacteria *Synechococcus elongatus* PCC 7942, which heterologously expresses the proton-sucrose symporter *cscB*. Under conditions of osmotic stress, this *cscB*^+^ strain irreversibly secretes up to 85% of fixed carbon as sucrose [38]. This cyanobacterial host has been utilized in synthetic microbial consortia with a variety of microbes, including model strains (*E. coli*, *Bacillus subtilis*, and *S. cerevisiae*), soil bacteria (*Pseudomonas putida*, *Halomonas boliviensis*), and diazotrophs (*Azotobacter vinelandii*) to produce a variety of products without additional sugar input [39–42]. Autotroph-heterotroph coupling is not limited to cyanobacteria—binary culturing of the eukaryotic algae *Haematococcus pluvialis* with *B. subtilis* demonstrated a coupling of oxygenic photosynthesis with respiration, such that neither external CO_2_ nor O_2_ supplies were necessary, suggesting that some of the overhead costs associated with autotrophs could be reduced in a properly-configured production system [43]. Similarly, co-cultures of eukaryotic algae *Chlorella minutissima* with *E. coli* can increase neutral lipid production and improve biodiesel quality [44]. These studies facilitate the application of CO_2_-to-fuel technologies.

### Nutrient crossing feeding using companion strains

Interacting partners can be designed to maintain stable interactions through detoxifying inhibitory substances. For instance, *Dehalococcoides mccartyi* is an important bacterium involved in the bioremediation of chlorinated solvents, but its incomplete Wood–Ljungdahl pathway generates toxic CO [45]. Co-cultures with CO-consuming bacterium *Desulfovibrio vulgaris Hildenborough* as the companion can significantly support *D. mccartyi* growth and TCE degradation ability. In another example, l‐ascorbic acid (vitamin C) is produced via a two‐step fermentation process to reduce costs and increase product quality. In the second step, a mixed fermentation consisting of a production strain (*Ketogulonicigenium vulgare*) and a companion strain (e.g. *Bacillus* spp.) is used to convert l‐sorbose to 2‐keto‐l‐gulonic acid and ascorbic acid [46]. The interaction mechanisms between *K. vulgare* and the companion strain have been studied, and it was found that the companion strain secreted metabolites to support *K. vulgare* growth (including proteins, some amino acids, and other unknown substances) while *K. vulgare* can also inhibit growth of the companion strain by lysis of companion cells (thus both mutualism and amensalism exist in this artificial ecosystem).

## Division of long-step biosynthesis pathways among different species

**Fig. 4:**
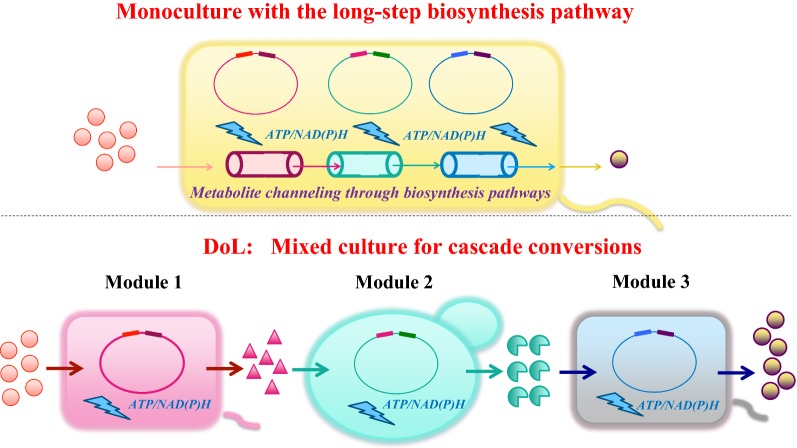
Schematic comparison of a microbial consortium and a monoculture in the expression of a challenging metabolic pathway. Microbial consortia-based division of metabolic pathways into multiple strains can reduce metabolic burden imposed on each individual constituent strain. Also, specific modules can be assigned to suitable strains for optimal enzyme expression (different colors of cells represent different host strains). However, DoL may break innate metabolite channeling and dilute intermediate metabolites in the biosynthesis pathways

The introduction of long-step biosynthetic pathways in a single bacterial strain can cause severe metabolic burden due to the overwhelming consumption of cellular build blocks and ATP for enzyme synthesis. In contrast, dividing the pathway into multiple strains in a synthetic consortium can split the overall metabolic burden among the constituent strains, with each strain bearing significantly lower stresses [21] (Fig. 4). Such a strategy has been adopted for the expression of an entire anthocyanin pathway from tyrosine in a four-strain consortium, employing an efficient tyrosine-producing strain as the first node strain [18]. This artificial microbial community gave rise to the direct production of ~ 10 mg/L pelargonidin 3-*O*-glucoside from glucose, which is functionally impossible via monoculture. Another advantage of microbial consortia is that the use of multiple species in one pathway can ensure that all genes are properly expressed [47]. In the microbial production of natural products, some genes with plant or other eukaryotic origins may not be functionally expressed due to codon bias, improper protein folding, or the lack of post-translational modifications [48]. Additionally, the effective operation of many modules relies on a sufficient supply of cofactors (such as NAD^+^/NADH and NADP^+^/NADPH) or active building blocks (such as acetyl-CoA and malonyl-CoA). While it is difficult to maximize every module simultaneously in a single strain, DoL can facilitate the functional expression of the complete pathway. With all the modules fully potentiated and the inter-module interference minimized by spatial segregation, DoL greatly improves pathway efficiency. A successful demonstration of such a method is the high-titer production of oxygenated taxanes in a *S. cerevisiae*–*E. coli* consortium. *S. cerevisiae* was used to express a P450 taxadiene hydroxylase (which could not be expressed functionally in *E. coli*), and *E. coli* was engineered for efficient taxadiene production. This co-culture generated 33 mg/L oxygenated taxanes, which were undetectable in either *E. coli* or yeast monoculture harboring the entire pathway [15]. Division of challenging pathways in optimal constituent strains in a microbial consortium is feasible for the bioproduction of various complex natural compounds such as flavonoids, isoprenoids and alkaloids. Moreover, DoL potentially simplifies optimization of these metabolic pathways. For a monoculture, construction of an efficient heterologous pathway usually involves adjustment of pathway components, such as promoters, ribosome binding sites, terminators, and vectors, followed by the Design-Build-Test cycle [49], which is labor-intensive and cost-ineffective. In comparison, the optimization of microbial consortia is somewhat easier, and is achieved simply by changing the ratio of the constituent strains [16]. Theoretically, the alteration of the culture component can be performed at any stage of the fermentation process by supplementing the desired subcultures.

## Limitations of DoL and future directions

Culturing microbial workhorses as a community faces several key limitations. First, many microbial community systems are dynamic and cannot achieve long-term production stability. Applications of consortia systems require identifying both an optimal inoculation ratio and an ideal product induction time. Nevertheless, the stability of the co-culture is expected to decrease in large volume and long-term fermentation processes due to spatiotemporal dynamics inside of reactors [24], incompatible growth requirements (e.g., the cross-feeding is insufficient to sustain net growth of both partners), or metabolite dilutions [50]. Second, two different species in co-cultures have to grow under suboptimal or compromised conditions. For example, to stabilize a co-culture of *E. coli* and *S. cerevisiae*, *S. cerevisiae* must be grown with acetate to avoid producing toxic ethanol [15]. Since acetate is not a favorable carbon source for yeast, the final bio-manufacturing capability is impaired. Third, metabolite transport can be an issue if a pathway is divided, such that the first product must be transported into the second host for further conversion. If the first product (e.g., a phosphorylated molecule) is unstable or unable to cross the cell membrane, or the second host lacks a transporter to uptake the precursor from the first host, the DoL will fail. Engineering effective transporters and efflux systems is not easy and may also add metabolic burdens [51]. Furthermore, long-step biosynthesis pathways often require proximity channeling to overcome in vivo diffusion limitation, metabolite loss, and thermodynamic barriers. DoL of a biosynthesis pathway between two different species may break innate channels and dilute intermediate concentrations, which unavoidably hinders biosynthesis efficiency [52] (Fig. 4). Fourth, microbial species may naturally evolve into distinct phenotypic subpopulations (e.g., *E. coli* may form two distinct cell populations with acetate cross-feeding) [53]. In mutualistic consortia, cheaters (individual cells that do not help the other species) can be observed—cheaters face less metabolic burden and can achieve a faster growth rate, but when they become dominant, the co-culture will be unable to survive [54]. For example, in a CBP-based isobutanol fermentation, fungus *T. reesei* and engineered *E. coli* consortia form stable cooperator–cheater dynamics [31]. Interestingly, even in a pure culture of synthetic hosts, nongenetic cell-to-cell variations can be high, allowing low-production cheaters to eventually become dominant [55].

Current community-based biomanufacturing mainly leverages natural systems and focuses on utilization of recalcitrant or cheap feedstock. In these desired platforms, metabolic interactions (e.g., cross feeding) can be robustly evolved. Still, there are few industrial applications based on the division of biosynthesis pathways among different species. For future DoL developments, a few approaches are promising. First, biosensors can be used to monitor and control cell sub-populations. In addition to quorum sensing for population monitoring, an RNA riboswitch-based biosensor module has been developed to provide real-time screening for overproducing cells in co-cultures [56]. Biosensor-regulator systems may be used for in vivo population quality control to continuously select for high-performing variants by suppressing the growth of cheaters [55]. Second, state-of-the-art bioreactor operations with the aid of readily-available high-throughput tools and real-time detection systems allow for feedback control/optimization of population composition (supplements of desired culture) and bioprocess parameters (e.g., substrate feeding) to maintain co-culture fermentation for stable and efficient biomanufacturing [21]. Third, the integration of non-bioprocess methods (Chem + electrobio) is another future direction [57]. Microbial electrosynthesis is a novel technology which combines metabolic engineering and electrochemistry within a biological reactor and has the ability to achieve 100% carbon efficiency by generating reducing equivalents (in the form of NADH) from electrons [58]. This type of electrochemical bioreactor may also capture and reduce CO_2_, thus moving carbon into the desired metabolic pathway. A similar concept has been used to combine hybrid inorganic and biological organisms for solar-to-chemical production [59, 60].

## Analysis and modeling of consortia and metabolic interactions between species

There are many tools being developed to study consortia and DoL applications. Kinetic modeling is the most common method to analyze nutrient cross-feeding and microbial interactions [20]. For example, *E. coli* and photoheterotrophic *Rhodopseudomonas palustris* cross-feed carbon (organic acids) and nitrogen (ammonium) to form a stable co-culture. Monod-based differential equations have been developed to reveal fermentation conditions for stabilizing mutualism [61, 62]. The model results indicate that organic acids from *E. coli* can be inhibitory and thus decrease carbon utilization efficiency and population equilibrium. For molecular-level understanding, functional genomics and high-quality metabolic modeling are needed to decipher cellular regulatory networks and integrated functions. For consortia, functional analysis can rely on typical microbiome tools, including targeted cell population analysis (such as 16S rRNA), metagenomic sequencing (DNA is recovered in an untargeted manner), metatranscriptomics (study the gene expression of microbial communities), and metaproteomics (study proteins expressed by microbiota to gain insight into functional potential). Microfluidic cell sorting can assist in the separation of individual species to study their DNA–RNA–Protein profiles under a community system. Moreover, measurement of metabolites within community systems can monitor the global community outcome and provide a snapshot of the general physiological state of microbes [46, 63].

To date, methods applicable for analyzing metabolic fluxes between two species (especially for two engineered strains derived from the same parent strain) are still rare. Currently, genome-scale models (GSMs) predict the feasible fluxes based on stoichiometry of the metabolic reactions, cellular objective functions, and constraints [64–68]. Co-culture/tri-culture stoichiometric models have been developed to analyze compositions of microbial communities for the biogas process [69]. Such models can potentially combine gene expression data or translational profiles as reaction constraints to capture information passage from DNA → RNA → proteins [70]. For example, GSM and metatranscriptomic data are used to study the uncultured bacterial symbiont “*Candidatus Endobugula sertula*” and identify their metabolic deficiencies [71]. Flux balance analysis (FBA) using reconstructed metabolic networks was attempted for the syntrophic *Desulfovibrio vulgaris/Methanococcus maripaludis* chemostat system [19]. The modeling results were compared to their continuous bioreactor data on lactate cultures in the absence of sulfate, and were found to accurately predict ecologically-relevant characteristics and growth parameters of bacterial communities. FBA, however, requires the assumption of a pseudo steady-state system. To capture complex dynamics in time and space of microbial communities, dynamic flux balance analysis (dFBA) is employed. In a co-culture model for cellulolytic *Clostridium cellulolyticum* and the solventogenic *Clostridium acetobutylicum*, an adapted dFBA was used where the key parameters required are k_m_ and V_max_ for cellulose solubilization and cell metabolism, as well as starting biomass concentrations for the two co-culture strains to set the input conditions for the growth dynamics [72]. dFBA can simulate the time-step interaction of the individual steady state models, and measurements of key central metabolites can be used to test the accuracy of the co-culture simulation. In another example, dFBA for multiple organisms was developed to address whether ecosystem-level behavior of structured communities can be predicted. The modeling framework with experimentally-confirmed species ratio data simulated the community behavior and metabolic interplay among two or three species inside of a colony [24]. This result can be integrated with economic analysis for a priori estimation of synthetic co-culture performance using cellulosic sugars and flue gas [73]. In parallel, new algorithms are proposed for designing synthetic microbial communities with desired features [74, 75]. Recently, the constraint-based modeling approach was applied to the human gut microbiome, resulting in a collection of 773 GSMs for gut-associated microorganisms [76]. In addition to the computational advances for modeling multi-species systems, other research has sought to expand the depth of modeling to include additional molecular details. An example of these computational advances is a modularized approach using multiple mathematical approaches to model the whole cell function of *Mycoplasma genitalium* [77]. Another significant study was the implementation of transcriptional and translational machinery into constraint-based models to predict gene expression levels [78]. Together, advances in single organism models and approaches for modeling microbial consortia will enable improved understanding of consortia dynamics and the ability to prospectively design and control consortia function.

^13^C-metabolic flux analysis (MFA) can measure intracellular carbon and energy fluxes. As a simple approach, MFA can treat co-culture as a single system and determine the bulk fluxomes of this system using traditional ^13^C-MFA approaches via labeling in protein-derived amino acids. This bulk MFA only describes the system as a whole, without metabolic exchanges. The full separation of metabolites from individual species is required to reveal subpopulation fluxes and interactions between two species. Separation of subpopulation is technologically difficult, even with fast cell-sorting. However, a “reporter protein” synthesized by a specific cell type can store ^13^C-fingerprints to investigate the microbial community. For mixed culture MFA, instead of detecting the ^13^C labeling patterns in amino acids from the total cellular protein, ^13^C patterns in amino acids from the reporter proteins can provide labeling information for each individual species. For example, photosystem I is a reliable reporter protein to probe symbiotic interactions in algae-heterotrophic bacterial communities [79]. Specifically, photosystem I proteins are abundant in algae and these proteins form large complexes that can be separated from the culture via ultracentrifuge and used as the reporter to study algae-bacteria cultures. For engineered species, the reporter protein can be overexpressed with a His-tag, allowing affinity purification from bulk culture [79, 80]. For example, the recombinant fusion glutathione S-transferase and green fluorescent protein can be used as the reporters to study mixed-culture of *E. coli* mutants. The reporter method quantitatively resolved the expected mutant-specific phenotypes down to subpopulation fractions of ~ 1% [81]. Similarly, peptide-based ^13^C-MFA has been developed to measure intracellular metabolic fluxes and inter-species metabolite exchange for complex microbial communities. Peptide identity and labeling patterns can be obtained in a high-throughput manner from modern proteomics techniques, which can recover metabolic fluxes in the same way as through the standard amino acid-based ^13^C-MFA [82]. Another approach for co-culture ^13^C-MFA is proposed by the Antoniewicz group, which determines metabolic flux distributions without the need for physical separation of cells or species-specific products via measurement of isotopic labeling of total biomass and elegant mathematical calculations. This approach can simulate both fluxes and the relative population size of each species in a mixed culture as well as inter-species metabolite exchange [83].

## Conclusion

Division of labor via mixed cultures can theoretically reduce metabolic burden to enable a system to utilize recalcitrant feedstock or generate products which require long-step heterologous pathways. However, microbial consortia represent complex and dynamic systems that are difficult to operate. Community based strain engineering, metabolic modeling, and flux analysis tools are rapidly developed recently, which may facilitate the wide application of DoL concept for biomanufacturing.
